# Systematic hyperparameter analysis of GRU and LSTM across demand pattern types: a demand-characteristic-driven meta-learning framework for rapid optimization

**DOI:** 10.1038/s41598-025-31508-x

**Published:** 2025-12-25

**Authors:** Ahmed O. El-Meehy, Amin K. El-Kharbotly, Mohammed M. El-Beheiry

**Affiliations:** Department of Design & Production Engineering, Faculty of Engineering, Ain Shams University, Cairo, Egypt

**Keywords:** Demand forecasting, Deep learning, Hyperparameter tuning, Demand characteristics, Integrated forecasting framework, Multi-period forecasting, Engineering, Mathematics and computing

## Abstract

Deep Learning (DL) offers powerful tools for demand forecasting by capturing complex nonlinear patterns and adapting to dynamic market conditions. Accurate forecasts are vital for optimizing production planning, reducing costs, aligning with customer demand, and efficient resource allocation. Forecast accuracy depends heavily on both dataset characteristics and DL hyperparameters, which influence model complexity and learning behavior. Although research efforts are focused on using data properties in demand classification and hyperparameter tuning for better DL accuracies, the efforts exerted in analyzing their impacts are few. This paper investigates how demand characteristics, such as variability, zero demand frequency, and spikiness, and DL hyperparameters of Gated Recurrent Unit (GRU) and Long Short-Term Memory (LSTM) affect multi-period forecast accuracy. Three types of demand patterns are analyzed: smooth demand, erratic demand without spikes, and erratic demand with spikes. Demand Complexity Index (DCI) is proposed as an integrated metric of demand characteristics, including demand variability, the amount of zero demands, and the degree of spikiness of the demand. To handle zero-demand periods and normalize accuracy across datasets, Weighted Mean Absolute Percentage Error (WMAPE%) is used to assess forecasting accuracy. Results show that the Coefficient of Variation (CV) is the most influential data feature, while Learning Rate is the most impactful hyperparameter affecting forecast accuracy. Demand complexity significantly influences forecasting accuracy, with WMAPE increasing by up to 14.6% per unit rise in DCI for GRU and 11.3% for LSTM, highlighting the need for complexity-driven model optimization. The main contribution of this work is introducing an integrated framework to tailor hyperparameter selection to input demand characteristics, enabling improved accuracy and faster processing.”

## Introduction

Deep Learning (DL) offers significant advantages in demand forecasting, including improved accuracy and adaptability to dynamic environments. Accurate forecasts are critical for production planning, cost control, resource allocation, and customer satisfaction. However, forecast accuracy depends heavily on both the characteristics of the demand data and the hyperparameters of DL models, which influence model complexity, generalization, and learning behavior.

Forecasting is a foundational decision in production planning. Overestimating demand leads to excess inventory costs, while underestimating results in missed opportunities. Accuracy errors may stem from the forecasting method itself or its compatibility with the data. Traditional methods like exponential smoothing require careful coefficient tuning, while DL methods demand precise hyperparameter selection. Moreover, data characteristics, such as variability, zero demand frequency, and spikiness, affect the suitability of forecasting techniques.

Understanding which DL hyperparameters and demand characteristics most influence forecast accuracy can guide practitioners in selecting appropriate configurations and methods. This is especially relevant in retail, where demand often includes zero values and spikes due to promotions or discounts.

DL forecasting methods are becoming more popular nowadays, yet the first question facing researchers and practitioners is how to select hyperparameters. This study presents a framework for researchers and practitioners to select DL hyperparameters and link this selection to demand characteristics. The proposed data-driven framework utilizes ML (Random Forest) to select GRU hyperparameters, and then the forecasts are established using GRU. The study’s main hypothesis is that for each dataset, there are hyperparameters that may minimize the forecasting error. Based on this hypothesis, the study aim is set to investigate the influence of deep learning hyperparameters and demand characteristics on the accuracy of forecasting the demands of multiple future periods (multi-period forecasting), fixed in this research at 24 periods, by employing feature regression and feature importance techniques to uncover correlations between forecast performance, data characteristics, and model settings. Through this analysis, the study seeks to derive practical insights that inform the selection of hyperparameters to enhance forecasting outcomes. This work systematically evaluates DL (GRU and LSTM) hyperparameters and demand characteristics using regression and correlation analysis. It identifies the most influential parameters affecting WMAPE% accuracy and offers practical guidance for tuning DL models. While focused on GRU and LSTM, the analytical framework is broadly applicable to other DL models and time series forecasting tasks.

The remainder of this paper is organized as follows: Sect. 2 presents a literature review covering forecasting techniques, the role of hyperparameters in ML and DL models, demand characteristics, and accuracy measures. Section 3 outlines the experimental design, including the forecasting models used, dataset descriptions, and selected hyperparameter settings. Section 4 provides the results and the experimental analysis. Section 5 discusses the results by investigating successful and failure forecasting situations, then introduces the integrated forecasting framework. Finally, Sect. 6 concludes the paper, and Sect. 7 offers future research recommendations.

## Literature review

### Forecasting methods

Machine learning tools for demand forecasting can be broadly categorized into two groups. The first includes kernel-based methods as Support Vector Regression^1^ or tree-based methods, such as Decision Tree^2^, Random Forest^3^ and Boosting methods, including Gradient Boosting^4^, Extreme Gradient Boosting^5^, LightGBM^6^ and CatBoost^7^.

The second category includes Deep Learning methods, particularly Recurrent Neural Networks (RNN), which are widely used for their ability to model sequential data and manage memory over time^8,9^. Common RNN variants in forecasting are the Gated Recurrent Unit (GRU)^10^ and Long Short-Term Memory (LSTM)^11^. GRU, introduced by^12^, simplifies LSTM’s structure and addresses long-short dependency issues like vanishing and exploding gradients. However, no consistent evidence favors one over the other. Additionally, Prophet^13^, Transformer-based models^14,15^ (as Informer^16^, FEDformer^17^, DLinear^18^, and PatchTST^19^, and TCN^20^ models are gaining traction in recent literature.

### Hyperparameter optimization

Machine Learning and Deep Learning have enabled the forecasting of complex demand patterns by capturing nonlinear correlations. However, they face two key challenges: the need for careful hyperparameter selection^8^, and the difficulty in interpreting these parameters. Common selection techniques include iterative search methods like GridSearchCV^3,21^, Random Search^22^, and Bayesian Optimization^23^. Advanced approaches involve Genetic Algorithms^24,25^, SEARCH^26^, HBO (Heap-Based Optimizer)^25^, and PSO^27^. Researchers have also explored loss functions to improve model learning: modified MSE with kernel skills^14,28^ triplet loss for power grid prediction^29^, and focal loss for detecting abnormal demand^30^. Despite these efforts, few studies have examined which hyperparameters most significantly affect forecast accuracy and to what extent.

### Demand classification metrics

Demand characteristics significantly influence the choice of forecasting methods. Classification based on ADI (zero-demand frequency) and variability of non-zero values into smooth, erratic, intermittent, and lumpy is now a common practice^31,32^. In retail and distribution contexts, even smooth or erratic products often show zero demand due to data granularity, which can affect ML and DL model behavior. Additionally, promotions may cause sporadic demand spikes. While smooth demand has been widely studied, erratic^33^ and sporadic types^34,35^ are less frequently addressed in isolation.

Demand classification relies on three statistical measures: Coefficient of Variation (CV), Average Demand Interval (ADI), and Peak-to-Mean Ratio (PMR). CV, the ratio of standard deviation to mean, reflects demand variability, while ADI, calculated as total periods divided by non-zero demand periods, indicates the frequency of zero demands^31^. PMR, commonly used in environmental^36,37^ and signal processing fields^38^, measures spikiness as the peak value over short periods divided by the mean. In this study, PMR helps assess the severity of sporadic demand.

### Multi-period forecasting

Multi-period forecasting is widely applied in non-industrial fields such as energy^39,40^, weather^41,42^, hydrology^43,44^, and finance^45–47^, where short-term predictions support operational decisions. For example^48^, proposed an attention-based parallel skip LSTM model for forecasting in energy, retail, and traffic, emphasizing the importance of multi-period forecasting in cyclical or seasonal data. Despite its relevance, this approach remains underexplored in industrial and supply chain contexts.

### Forecast error metric

This paper investigates how data properties and deep learning hyperparameters affect multi-period forecasting accuracy across datasets that include zero-demand periods. To enable comparison across datasets with varying statistics and to handle zero demands effectively, the Weighted Mean Absolute Percent Error (WMAPE%)^49^ is used. WMAPE%, calculated as the sum of absolute errors divided by the sum of actual demands, provides a normalized percentage metric that avoids the division-by-zero issue found in MAPE^49^.

The findings of the literature are summarized in Table 1. Despite the achieved advancements in DL forecasting and hyperparameter optimization, essential knowledge gaps still limit practical implementation. prior studies employ methods as GridSearch, genetic algorithms, and Bayesian optimization, yet seldom explore which hyperparameters drive forecast accuracy or how their effects alter with data characteristics. Furthermore, CV, ADI, and PMR are recognized demand classifiers; however, their interactions with DL hyperparameters remain largely unexplored, which forces practitioners to rely on costly iterative tuning for new demands. Besides, existing literature relies on successful forecasts, showing limited analysis of failure cases and how they vary across smooth, erratic, and spike-prone patterns.

**Table 1:** Key findings drawn from the literature review, including forecasting methods, hyperparameter optimization, and demand classification.

Area	Key findings	Methods/Techniques	Research gaps
Forecasting methods	DL methods (GRU, LSTM, Transformers) are effective for sequential data; GRU simplifies LSTM without clear superiority	RNN variants, Transformer-based (Informer, PatchTST), TCN	No consensus on which architecture is universally better
Hyperparameter optimizations	Common techniques: GridSearch, Random Search, Bayesian Opt, Genetic Algorithms	Iterative search, metaheuristics (HBO, PSO), modified loss functions	Few studies examine which hyperparameters most affect accuracy and to what extent
Demand classification	CV, ADI, PMR characterize demand patterns (smooth, erratic, intermittent, lumpy); zero-demands and spikes are common in practice	Statistical metrics (CV, ADI, PMR) for classification	Erratic and sporadic demand types are less studied; PMR is underutilized for spike characterization
Multi-period forecasting	Widely used in energy, weather, and finance; critical for cyclical/seasonal data	Attention mechanisms, parallel architectures	Underexplored in industrial/supply chain contexts
Error metrics	WMAPE% handles zero-demand periods better than MAPE	WMAPE% avoids division-by-zero issues	Limited comparison across diverse demand types

This study addresses these gaps by systematically quantifying DL (GRU and LSTM) hyperparameter effects across various demand types using controlled experimentation (Latin Hypercube sampling and OFAT on extracted data from M4 and M5 competitions). It provides empirical links between demand characteristics and optimal hyperparameter configurations. To introduce these insights for practitioners, this study introduces an integrated forecasting framework that predicts hyperparameters directly from data characteristics to shift tuning from an expensive to a rapid search task.

## Experimental design

This study investigates whether demand data characteristics or DL hyperparameters influence forecast accuracy. The following subsections outline the data classification criteria, selected hyperparameters, and experimental design.

### Data description

The datasets are categorized into three types, smooth, erratic without spikes (EwoS), and erratic with spikes (EwS), based on three key metrics commonly used in demand data classification for time series forecasting applications. The Coefficient of Variation (CV), defined in Eq. (1) as the ratio of standard deviation to mean, measures demand variability; the higher the CV, the higher the variability in demand data. A CV² (square of Coefficient of Variation) threshold of 0.49 is used to distinguish smooth (< 0.49) from erratic (> 0.49) demand. The Average Demand Interval (ADI), given in Eq. (2), reflects the frequency of zero-demand periods and is calculated as the total number of periods of demand data divided by the number of non-zero demands, with values ranging from 1 (no zeros; because the total number of demand points equals the number of non-zero demands within this dataset) to 1.32 (after which the demand is considered intermittent or lumpy demand). Also, the higher the ADI, the higher the fluctuation of demand data, as it means demand is being alternated between zero and non-zero demands more frequently. Lastly, the Peak-to-Mean Ratio (PMR), defined in Eq. (3), assesses spikiness, where a PMR greater than 10 indicates spiky demand. The PMR is the ratio between the maximum peak in demand data and the mean of the same data; the higher the PMR, the spikier the data is.

1 $$\:CV=\frac{\sigma\:}{\mu\:}$$

2 $$\:ADI=\frac{Total\:Periods}{Total\:Demand\:Buckets}$$

3 $$\:PMR=\frac{Max}{Mean}$$

Table 2 presents key statistics for each dataset, including total demand points, zero/non-zero counts, 0%, and basic descriptive metrics (min, mean, max, median, range, MAD, and standard deviation). The most critical indicators for classification are CV², ADI, and PMR. Smooth datasets show low CV² and PMR values, while erratic datasets exceed the CV² threshold and are further split based on PMR into spiky and non-spiky categories. All datasets are extracted from the M4 and M5 competitions data^50,51^. For each dataset, the training set is set as all the data except for the forecasting horizon (test set), set as the last 24 periods. The forecasting horizon is set with this value since it is more practical, suitable for planning purposes, and medium-term planning purposes.

**Table 2:** Datasets classification and statistics.

Dataset	Data type	Count	Non-zero Count	Zero Count	0%	Min	Max	Mean	Std Dev	MAD	ADI	CV^2^	PMR
SS1	Smooth	1913.0	1909.0	4.0	0.0	0.0	3800.0	1544.5	476.2	369.5	1.0	0.1	2.5
SS2		1913.0	1881.0	32.0	0.0	0.0	1650.0	530.8	265.3	206.8	1.0	0.2	3.1
SS3		1913.0	1788.0	125.0	0.1	0.0	1500.0	349.8	203.8	158.7	1.1	0.3	4.3
SS4		1913.0	1518.0	395.0	0.2	0.0	1750.0	462.5	322.4	263.2	1.3	0.5	3.8
SS5		1913.0	1893.0	20.0	0.0	0.0	2600.0	732.0	313.9	245.5	1.0	0.2	3.6
E8	EwoS	1913.0	1564.0	349.0	0.2	0.0	800.0	152.6	130.1	101.4	1.2	0.7	5.2
E3		1913.0	1522.0	391.0	0.2	0.0	1400.0	208.3	189.7	147.0	1.3	0.8	6.7
EE1		1913.0	1776.0	137.0	0.1	0.0	1750.0	381.7	291.0	226.5	1.1	0.6	4.6
E10		1913.0	1542.0	371.0	0.2	0.0	4600.0	590.6	616.9	470.2	1.2	1.1	7.8
E9		1913.0	1515.0	398.0	0.2	0.0	5200.0	594.5	700.0	508.5	1.3	1.4	8.7
E5	EwS	1913.0	1677.0	236.0	0.1	0.0	5600.0	393.1	442.4	299.5	1.1	1.3	14.2
EE2		1913.0	1470.0	443.0	0.2	0.0	3450.0	359.7	485.6	326.8	1.3	1.8	10
E7		1913.0	1521.0	392.0	0.2	0.0	14700.0	494.1	695.2	433.4	1.3	2.0	29.8
EE4		1913.0	1474.0	439.0	0.2	0.0	5500.0	244.8	406.0	221.3	1.3	2.7	22.5
EE3		1913.0	1680.0	233.0	0.1	0.0	31700.0	215.8	745.7	155.9	1.1	11.9	146.9

### Forecasting models

In this research, a Gated Recurrent Unit (GRU) model and a Long Short-Term Memory (LSTM) are adopted as examples of DL methods to forecast time-series data due to their ability to capture sequential data. The model architecture and configurations are adapted to allow for predicting multiple periods ahead.

For both models, Data preprocessing involved setting a fixed random seed (42) for reproducibility, removing missing rows, and converting date fields to a datetime format. Temporal features such as year, month, and day were extracted to capture date-related effects, and a counter feature was added to represent elapsed days. All input features were normalized to the [0,1] range to ensure model stability and faster convergence. Sliding windows of recent observations were constructed for each forecast horizon, and all features were combined into a multi-feature input for the adopted DL models. Post-processing included inverse scaling to restore original demand values and rounding predictions to integer quantities.

#### Gated recurrent unit (GRU)

**Fig. 1:**
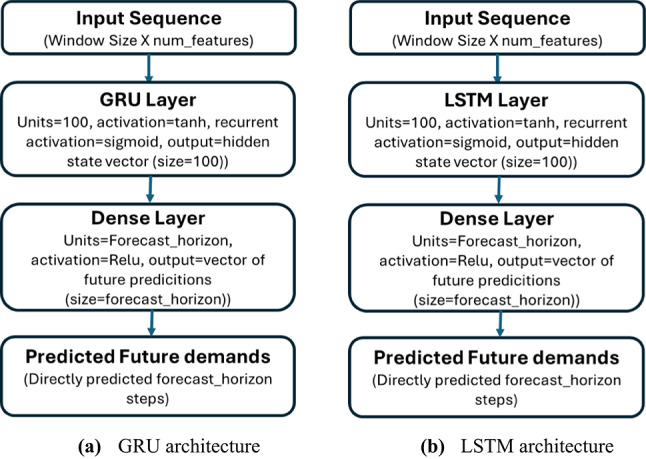
The architectures of the adopted GRU and LSTM models.

The GRU model, displayed in Fig. 1(a), consists of a GRU layer (100 units, tanh activation, sigmoid recurrent activation) and a Dense output layer with ReLU activation. This configuration was selected after testing all combinations of Sigmoid, ReLU, and tanh on sample datasets for stability. The model uses MSE as the loss function and Adam optimizer for adaptive learning rate control. Early stopping is not applied in this model to avoid ending the learning process without finishing the exact epoch values to be then analyzed. In addition, the datasets used are selected to be relatively large to reduce the error of overfitting.

#### Long Short-Term memory (LSTM)

To enforce the generalization of the findings of GRU forecasting to DL forecasting methods, all the experimentation conducted with GRU is also made with LSTM for comparison. The LSTM model is also a direct multi-step forecasting model, and the preprocessing and post-processing steps of the LSTM are similar to those of the GRU for fair comparison. The input layer is defined by the window size and the input feature. Then an LSTM layer is used with 100 units, ‘tanh’ activation, and sigmoid recurrent activation. After this, a Dense output layer with units equal to the forecast horizon and a ‘ReLU’ activation, produces all future steps. Adam optimizer and MSE Loss are adopted. The LSTM model’s architecture is shown in Fig. 1(b).

### Hyperparameters

This study focuses on the key GRU and LSTM hyperparameters commonly cited in the literature, along with a synthetic parameter, Window Size, to enhance multi-step forecasting by learning from sequential patterns. The selected hyperparameters and their ranges, along with the justification of these ranges, are shown in Table 3:

**Table 3:** Selected ranges of hyperparameters and the justification of the selection of these ranges.

HP	Range	Description	Justification
Batch Size (BS)	5–100	Number of training samples per update; affects memory usage and training speed.	Smaller sizes (5–30) allow frequent updates and low memory use; larger sizes (70–100) speed up training but require more memory.
Window Size (WS)	10–100	Number of past time-steps used as input.	Captures short to medium-term dependencies; value 24 helps detect potential symmetric patterns.
Learning Rate (LR)	0.0001–0.1	Controls weight updates during training.	Lower values (0.0001–0.01) ensure stable convergence; higher values (0.05–0.1) accelerate training but risk instability.
Epochs (EP)	10–100	Number of full passes through the dataset.	Lower counts (10–30) reduce overfitting; higher counts (60–100) support learning complex patterns.

### Forecast accuracy measure

To evaluate multi-period DL forecasts with occasional zero demands, Weighted Mean Absolute Percentage Error (WMAPE%) is used for its suitability. It assesses overall forecast accuracy across the horizon and handles zero actuals by summing all actual values before division. As shown in Eq. (4), WMAPE% is the ratio of the cumulative sum of absolute forecast errors of periods constituting the forecasting horizon to the sum of actual demands, expressed as a percentage. Because the planning horizon is relatively long, the values of the WMAPE% are expected to be large due to the accumulation of possible errors over the various periods.

4 $$\:WMAPE\%=100*\:\frac{\sum\:_{1}^{T}|{A}_{t}-{F}_{t}|}{\sum\:_{1}^{T}{A}_{t}}$$

### Hyperparameter experimental values

A hybrid DOE is adopted in this research, combining two complementary approaches:

*Latin Hypercube Sampling (LHS)*:

To efficiently sample hyperparameter ranges, Latin Hypercube Sampling (LHS) is used. It divides each parameter’s range into equally probable intervals and randomly selects one unique value from each, ensuring broad and even coverage with a manageable number of samples. The resulting hyperparameter values are listed in Table 4.

**Table 4:** Hyperparameter values according to the Latin hypercube design of experiments.

Run	EP	BS	WS	LR	Run	EP	BS	WS	LR	Run	EP	BS	WS	LR
1	10	5	10	0.0001	11	80	5	100	0.05	21	10	100	24	0.001
2	50	8	18	0.0005	12	100	8	10	0.1	22	50	5	30	0.0001
3	80	16	24	0.001	13	10	50	18	0.0005	23	80	8	50	0.005
4	100	24	30	0.005	14	50	60	24	0.001	24	100	16	65	0.01
5	10	50	50	0.01	15	80	80	30	0.0001	25	10	80	100	0.05
6	50	60	65	0.05	16	100	100	50	0.005	26	50	100	10	0.1
7	80	80	85	0.1	17	10	24	85	0.01	27	80	24	18	0.0001
8	100	100	100	0.0001	18	50	50	100	0.05	28	100	50	24	0.0005
9	10	16	65	0.005	19	80	60	10	0.1	29	10	60	30	0.001
10	50	24	85	0.01	20	100	80	18	0.0005	30	50	80	50	0.005

*One-Factor-At-a-Time (OFAT)*

Used to isolate the effect of each hyperparameter while keeping the others as constant, and to ensure that the RF Hyperparameter prediction model captures non-linear and monotonic effects of hyperparameters. The hyperparameter values for these added experiments are listed in Table 5. The rationale behind the selection of the fixed values of the hyperparameters is as follows. The value of Epochs of 50 balances training with computational costs throughout the OFAT portion of the experimentation and provides sufficient training without overfitting. While the Learning Rate’s value was set to 0.001 based on preliminary experimentation, and showed significantly reliable stability in convergence for various data characteristics (CV, ADI, and PMR). The Window Size is fixed at the maximum value, 100, to maximize the available prior information for better recognition of sequential dependencies and to avoid inefficient studying of other hyperparameters’ individual effects due to insufficient historical information of lower WS values. A Batch of size 24 is a mid-range value that balances stochasticity with stability; besides, this value might create a natural homogeneity between the prediction targets and the learning batches. Moreover, it might simplify interpretation and practical implications when the forecasting horizon equals the batch size.

On the other hand, the other portion of DOE based on Latin Hypercube Sampling ensures wider coverage by assigning lower and higher values of epochs, learning rate, and batch size hyperparameters.

**Table 5:** Hyperparameter values according to the One-Factor-At-a-Time (OFAT) design of experiments.

Run	EP	BS	WS	LR	Run	EP	BS	WS	LR	Run	EP	BS	WS	LR
31	50	24	10	0.001	40	50	24	100	0.0005	49	100	24	100	0.001
32	50	24	18	0.001	41	50	24	100	0.001	50	50	5	100	0.001
33	50	24	24	0.001	42	50	24	100	0.005	51	50	8	100	0.001
34	50	24	30	0.001	43	50	24	100	0.01	52	50	16	100	0.001
35	50	24	50	0.001	44	50	24	100	0.05	53	50	24	100	0.001
36	50	24	65	0.001	45	50	24	100	0.1	54	50	50	100	0.001
37	50	24	85	0.001	46	10	24	100	0.001	55	50	60	100	0.001
38	50	24	100	0.001	47	50	24	100	0.001	56	50	80	100	0.001
39	50	24	100	0.0001	48	80	24	100	0.001	57	50	100	100	0.001

This combination ensures both comprehensive range coverage (LHS) and clear interpretability (OFAT), which is essential for training the Random Forest data-driven part of the integrated framework, which generalizes across various datasets.

Experiments were conducted across all three dataset categories: Smooth, EwoS, and EwS. Analyses were performed both per category and on the combined dataset. In cases where the model failed to forecast, producing zero demand across the horizon, those configurations were excluded from analysis to avoid misleading results. However, these failure cases are discussed separately to inform practical avoidance strategies.

### Analysis of results

To explore the relationship between data properties, DL hyperparameters, and forecasting error, particularly for smooth and erratic demands with zeros and spikes, the analysis is structured into three parts: linear correlation, non-linear correlation, and practical insights.

Before analyzing linear correlations, it is important to assess whether demand complexity influences forecasting accuracy. This is done by plotting WMAPE% against the Demand Complexity Index (DCI), a term introduced by the authors of this study, defined as the sum of the normalized CV, ADI, and PMR, as shown in Eqs. (5) and (6). It is assumed that the demand variability (measured with CV), the amounts of zeros in demand (measured in ADI), and the severity of spikes in demand data (measured in PMR) are major players in increasing the complexity of demand data, and hence, their high values might degrade the forecasting accuracy. However, describing the complexity of demands using multiple factors might be challenging in analysis and interpreting results. The DCI is a single factor combining these three measures, yet, normalized since their scales are significantly different. The normalization is made for a group of datasets or dataset characteristics under analysis. The minimum and maximum values of each measure is taken from the characteristics of these datasets.

5 $$\:{CV}_{norm}=\frac{CV-{CV}_{min}}{{CV}_{max}-{CV}_{min}},\:\:{ADI}_{norm}=\frac{ADI-{ADI}_{min}}{{ADI}_{max}-{ADI}_{min}},\:\:{PMR}_{norm}=\frac{PMR-{PMR}_{min}}{{PMR}_{max}-{PMR}_{min}}$$

6 $$\:Demand\:Complexity\:Index\:\left(DCI\right)={CV}_{norm}+{ADI}_{norm}+{PMR}_{norm}$$

To introduce the DCI as an index that replaces CV, ADI, and PMR, a multiple correlation (Pearson’s) test is conducted, showing significant, nearly perfect multiple correlation in Smooth, EwoS cases, and a very strong correlation in the case of EwoS.

The correlation of the DCI and the WMAPE% measured is analyzed using Pearson’s correlation coefficient and Spearman’s rank coefficient to address the linear and monotonic correlations between them. The results show significant correlations for both measures in all demand cases. However, the correlations are stronger in smooth and EwoS cases while they are weaker in the presence of demand spikes, as these spikes might be recognized by the DL methods as outliers, and the remaining data might be considered as smooth or EwS with lower WMAPE% values. This might also be the reason for the negative correlation appearing in this case. Table 6 shows the values of the two coefficients and their p-values for different demand cases, and the value of Pearson correlation for multiple correlation with demand characteristics, as well as its p-value.

**Table 6:** Pearson’s and spearman’s correlations between demand complexity index (DCI) and WMAPE%, and pearson’s multiple correlation between DCI and (CV, ADI, and PMR) for smooth, EwoS, EwS, all demand cases.

Problem	Correlation with WMAPE%				Multiple Correlation with (CV, ADI, PMR)	
Test	Pearson Correlation	Pearson *p*-value	Spearman Correlation	Spearman *p*-value	Pearson correlation	*p*-value
All	**0.3683**	~ 0	**0.6403**	~ 0	1.0	~ 0
Smooth	**0.7725**	~ 0	**0.8345**	~ 0	0.9997	~ 0
EwoS	**0.6166**	~ 0	**0.6832**	~ 0	0.9882	~ 0
EwS	**-0.2694**	~ 0.0003	**-0.5322**	~ 0	0.9355	~ 0

Linear relationships between WMAPE% and each feature (and between features) are assessed using the Pearson correlation coefficient (R). R is also used to show the correlation between each pair of the independent variables represented on a heatmap. While correlation matrices show pairwise relationships, VIF assesses overall multicollinearity by regressing each feature against all others. It’s calculated using Eq. (7). The (R^2^) in Eq. (7) is the coefficient of determination of an independent variable when regressed on all other independent variables.

7 $$\:VIF=\frac{1}{1-{R}^{2}}$$

Values between 1 and 5 indicate low to moderate multicollinearity; 5–10 suggest moderate to high; above 10 signal severe multicollinearity.

**Fig. 2:**
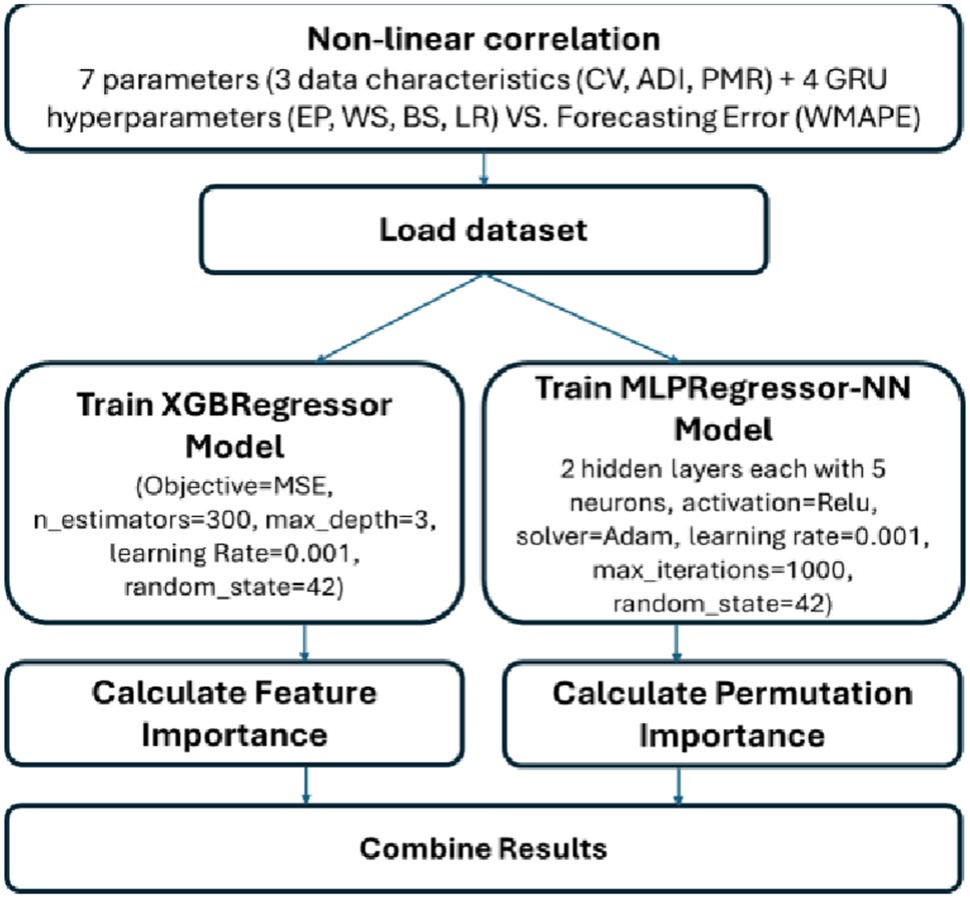
The integrated XGBoost and Multi-Layer Perceptron model serves as an indicator of the non-linear correlation between WMAPE% and the problem’s features.

As for feature importance, Neural Network (NN) and Extreme Gradient Boosting (XGBoost) are both used to rank the features according to their importance as determined by the two methods, as seen in Fig. 2. Both methods are used to ensure the importances of features using ML methods that can detect non-linear correlations. The XGBoost model is used to detect the feature importance based on feature importance determined by the weight of each feature (which counts the number of times a feature is used to split a decision tree). Model configuration (objective=’reg: squarederror’, n_estimators = 300, max_depth = 3, learning_rate = 0.001, random_state = 42). The NN model is a Multi-Layer Perceptron (MLP) model, which is used to determine the feature importance based on permutation importance (how much the error increases when the value of a single feature is randomly shuffled). Model configuration (Hidden layers: (5, 5), two hidden layers with 5 neurons each, activation function = relu, single neuron output layer, solver = adam, learning_rate_init = 0.001, max_iterations = 1000, random_state = 42, standardScaler is used in preprocessing).

## Results

The forecasting results are evaluated using WMAPE% as the dependent variable, with independent features drawn from both data characteristics (CV, ADI, PMR) and model configurations (Epochs, Batch Size, Window Size, Learning Rate). The analysis involves scatter plots to visually examine relationships between WMAPE% and dataset features, linear correlation assessments between WMAPE% and all variables, as well as among the variables themselves, and non-linear correlation modeling using XGBoost and Neural Networks. The findings are then summarized to highlight practical insights relevant to model performance and feature influence.

### Effect of demand characteristics on forecast accuracy

**Fig. 3:**
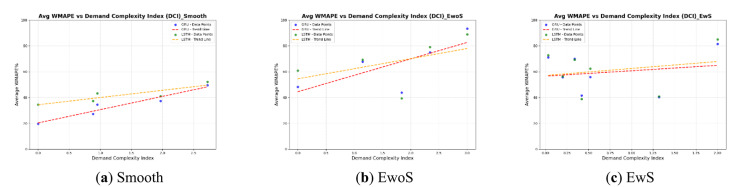
Scatter diagram of average WMAPE% values against data properties described by CV, ADI, and PMR in different demand cases for GRU and LSTM models (the increase in demand complexity increases WMAPE% on average).

Figure 3 shows scatter plots suggesting that data properties can influence forecast accuracy, even with DL models. In each of the three plots, the DCI (each point represents a single dataset) is drawn with the average WMAPE% of all successful forecasts. For Smooth and EwoS datasets, WMAPE% tends to increase with higher demand complexity resulting from higher variability, number of zeros, and spikiness. This trend is lower in slope for EwS as there are larger ranges for each of the demand characteristics, which reduces the values of the DCI. The reason for the lower slope in the EwS case might be that when the demand’s spikiness is relatively high, the GRU and LSTM models deal with the spikes as outliers and learn from the rest of the datasets, which might be less complex, i.e., smooth or EwoS. Although GRU generally achieves lower WMAPE than LSTM, the performance gap narrows as demand complexity (DCI) increases. For the aggregated dataset, GRU showed a stronger sensitivity to complexity, with WMAPE rising by 14.6% per unit increase in DCI (range: 19.66–93.23%), compared to 11.3% for LSTM (range: 34.59–88.80%). Within individual demand types, smooth patterns exhibited the lowest sensitivity (10.3% for GRU, 5.6% for LSTM), while erratic patterns without spikes showed higher impacts (12.7% and 7.8%, respectively). Erratic patterns with spikes had the smallest slopes (4.2% for GRU, 5.4% for LSTM), indicating that spike-driven variability affects both models differently. These findings confirm that as demand complexity increases, forecasting accuracy deteriorates, and GRU tends to be more affected than LSTM, emphasizing the need for complexity-aware model tuning.

### Linear relation between data properties and hyperparameters with forecast accuracy

#### Absolute (Individual) correlation with WMAPE%

Table 7 illustrates the linear correlation between WMAPE% and each feature across different dataset types for both GRU and LSTM methods. For GRU, overall, CV demonstrates a moderate correlation (~ 52%), while PMR and ADI show weak to very weak associations. In the Smooth and EwoS datasets, CV exhibits a strong correlation (exceeding 70%), with ADI and PMR having moderate influence. Notably, PMR is more impactful than ADI in the EwoS dataset, whereas ADI plays a more direct role in influencing WMAPE% in the EwS dataset. For LSTM, WMAPE% shows moderate correlation (~ 43%) with CV and weak correlations with PMR (~ 28%) and LR (~ 23%). For smooth demands, notably, the learning rate dominates the correlation with WMAPE% (~ 76%), while CV, ADI, and PMR exhibit weak correlations. CV and PMR have moderate correlations in the case of EwoS of values (~ 59%) and (44%), respectively. In EwS, and similar to what is seen with GRU, the correlation with CV is negligible, while it is moderate with ADI (~ 53%) and weak with PMR.

The observed positive correlation between WMAPE% and demand characteristics reflects fundamental forecast difficulty mechanisms. A high coefficient of variation (CV) indicates that variance dominates the mean, creating low signal-to-noise ratios where the model struggles to distinguish predictable patterns from random fluctuations. For intermittent demand patterns characterized by frequent zero periods, the forecasting task becomes a dual challenge: the model must accurately predict both the timing of non-zero demand occurrences and their magnitudes. Prediction errors on zero-demand periods disproportionately penalize WMAPE% while simultaneously providing weak gradient signals during training, hindering effective learning. High average demand interval (ADI) amplifies these effects by increasing the frequency of zero demands, further degrading information density in the temporal sequence. Peak-to-mean ratio (PMR), measuring the ratio of maximum to mean demand, quantifies the degree to which extreme outliers dominate the demand distribution. In high-PMR scenarios, forecast accuracy becomes critically dependent on spike prediction performance: a single missed spike event can contribute catastrophically to overall WMAPE%, as the absolute error from extreme values dominates the metric calculation.

**Table 7:** Absolute pearson’s correlation coefficients between WMAPE% and each of the problem features, for GRU and LSTM forecasting methods, with all demand cases.

	Smooth			EwoS			EwS			All		
	R	p-value	CI (95%)	R	p-value	CI (95%)	R	p-value	CI	R	p-value	CI (95%)
GRU												
CV	***0.724***	***~ 0***	***[0.65***,*** 0.78]***	***0.75***	***~ 0***	***[0.68***,*** 0.81]***	0.064	0.402	[-0.21, 0.09]	***0.523***	***~ 0***	***[0.46***,*** 0.58]***
ADI	***0.685***	***~ 0***	***[0.6***,*** 0.75]***	***0.485***	***~ 0***	***[0.37***,*** 0.58]***	***0.542***	***~ 0***	***[-0.64***,*** -0.43]***	***0.128***	***~ 0.002***	***[0.05***,*** 0.21]***
PMR	***0.644***	***~ 0***	***[0.55***,*** 0.72]***	***0.653***	***~ 0***	***[0.56***,*** 0.73]***	***0.268***	***~ 0***	***[0.12***,*** 0.4]***	***0.282***	***~ 0***	***[0.21***,*** 0.35]***
LR	0.075	0.297	[-0.07, 0.21]	0.079	0.27	[-0.22, 0.06]	0.069	0.364	[-0.08, 0.22]	0.063	0.125	[-0.14, 0.02]
EP	***0.21***	***0.003***	***[0.07***,*** 0.34]***	0.088	0.219	[-0.05, 0.22]	0.026	0.736	[-0.12, 0.17]	0.075	0.069	[-0.01, 0.15]
WS	0.041	0.565	[-0.18, 0.1]	0.065	0.365	[-0.2, 0.08]	0.028	0.713	[-0.18, 0.12]	0.035	0.394	[-0.12, 0.05]
BS	***0.03***	***0.682***	***[-0.17***,*** 0.11]***	0.039	0.586	[-0.18, 0.1]	0.017	0.82	[-0.17, 0.13]	0.012	0.769	[-0.09, 0.07]
LSTM												
CV	***0.335***	***~ 0***	***[0.22***,*** 0.44]***	***0.585***	***~ 0***	***[0.49***,*** 0.67]***	0.119	0.103	[-0.02, 0.26]	***0.428***	***~ 0***	***[0.36***,*** 0.49]***
ADI	***0.366***	***~ 0***	***[0.25***,*** 0.47]***	0.131	0.05	[-0.00, 0.26]	***0.53***	***~ 0***	***[-0.63***,*** 0.42]***	0.035	0.366	[-0.04, 0.11]
PMR	***0.261***	***~ 0***	***[0.14***,*** 0.37]***	***0.439***	***~ 0***	***[0.33***,*** 0.54]***	***0.369***	***~ 0***	***[0.24***,*** 0.49]***	***0.275***	***~ 0***	***[0.2***,*** 0.34]***
LR	***0.761***	***~ 0***	***[0.7***,*** 0.81]***	0.014	0.837	[-0.12, 0.14]	0.033	0.648	[-0.17, 0.11]	***0.228***	***~ 0***	***[0.15***,*** 0.3]***
EP	0.075	0.254	[-0.2, 0.05]	0.03	0.659	[0.00, 0.16]	0.045	0.5411	[0.10, 0.19]	0.002	0.951	[-0.07, 0.08]
WS	0.09	0.159	[-0.04, 0.21]	0.51	0.15	[-0.18, 0.08]	0.011	0.885	[-0.15, 0.13]	0.009	0.808	[-0.09, 0.07]
BS	***0.208***	***0.001***	***[0.08***,*** 0.32]***	0.016	0.812	[-0.15, 0.12]	0.086	0.211	[-0.06, 0.22]	0.07	0.671	[-0.01, 0.15]

#### Correlation matrix heatmap for the independent variables

**Fig. 4:**
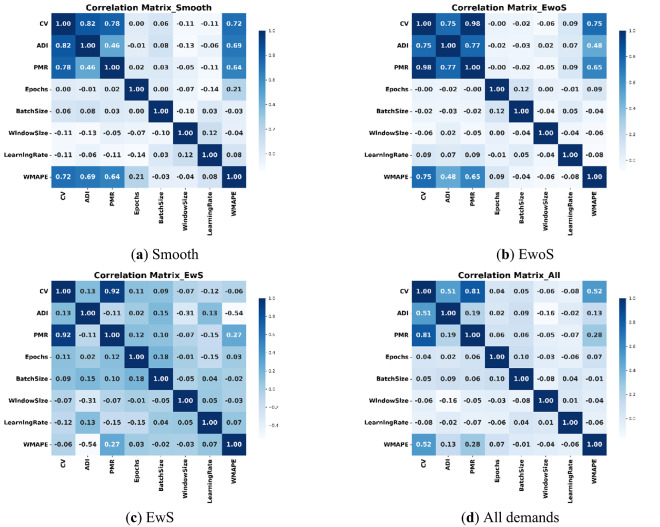
Correlation matrix heatmaps of independent demand characteristics and hyperparameter features for different demand situations (**a**) Smooth, (**b**) Erratic without Spikes, (**c**) Erratic with Spikes, and (**d**) All demand, with GRU model (demand characteristics show higher inter-correlations than GRU hyperparameters).

Figure 4 presents heatmaps illustrating the linear correlations among features for the GRU model. Overall, GRU hyperparameters exhibit minimal linear correlation with each other and with the data properties. In the full dataset, a strong correlation is observed between CV and PMR (0.81), while CV and ADI show a moderate correlation (0.51), and ADI and PMR are weakly correlated. In the Smooth datasets, CV demonstrates strong correlations with both ADI (0.82) and PMR (0.78). Notably, in the EwoS dataset, CV and PMR reach an exceptionally high correlation of 0.98, with ADI also showing strong associations with both parameters. Overall, data properties are interrelated, with PMR consistently showing the strongest correlation with CV, highlighting its role in demand variability.

**Fig. 5:**
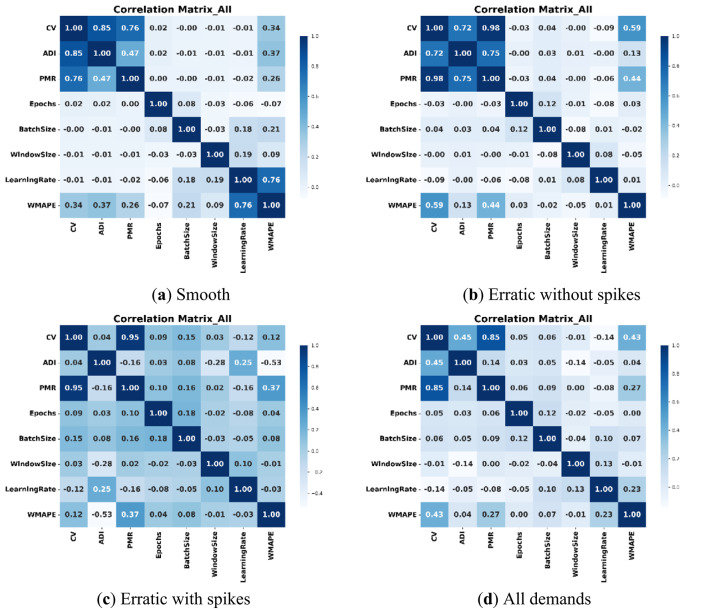
Correlation matrix heatmaps of independent demand characteristics and hyperparameter features for different demand situations (**a**) Smooth, (**b**) Erratic without Spikes, (**c**) Erratic with Spikes, and (**d**) All demand, with LSTM model (demand characteristics show higher inter-correlation than LSTM hyperparameters).

Figure 5 shows the correlation matrix heatmap for the independent variables in the case of LSTM forecasting. The values of correlations are almost the same as those of the GRU (Fig. 4). Some insights can be drawn from the results of both. First, the DL hyperparameters do not show recognizable correlations among each other or with the data characteristics parameters. Second, the PMR seems to be the dominant contributor to the value of CV, which indicates that the severity of peaks (PMR) in the demand data should be given attention, as it might have a significant impact on the variation of the data (CV). The impact of ADI on the value of CV is strong as well in cases of smooth and EwoS cases, yet it diminishes compared to the PMR when severe spikes are found in the data.

#### Variance inflation factor (VIF)

As shown in Table 8, GRU hyperparameters (Learning Rate, Epochs, Batch Size, Window Size) consistently have VIF < 5, indicating minimal multicollinearity. Data properties (PMR, ADI, CV) show high VIF values, especially in EwoS, suggesting strong interdependence and the need for joint analysis to avoid misleading interpretations. The relatively high values in the case EwoS are justified by the high correlation values found for this case in the correlation matrices (Figs. 4 and 5). For instance, in GRU and LSTM, the correlation between CV and PMR is close to 1 (~ 0.98) in both cases. This makes the denominator of the VIF, i.e., (1-R^2^) becomes a significantly small number that results in this high VIF.

The high collinearity between the three demand characteristic measures suggests that a single gathering metric that considers all their effects without duplication might be more practical and more concise in addressing demands.

**Table 8:** Variance inflation factors for different hyperparameters in different demand cases, for GRU and LSTM models.

VIF	Smooth		EwoS		EwS		All	
Feature	GRU	LSTM	GRU	LSTM	GRU	LSTM	GRU	LSTM
CV	***49.4***	***42.8***	***845.4***	***731.9***	***62.4***	***68.6***	***25.2***	***27.3***
ADI	***57.2***	***58.8***	***185.8***	***171.8***	***19.5***	***19.6***	***18.8***	***18.5***
PMR	***63.2***	***59.6***	***322.2***	***275.3***	***12.2***	***16***	5.4	6.6
EP	4.9	5	5	5.1	5.1	5.1	4.4	4.5
BS	2.9	3	2.9	2.9	3.1	3	2.8	2.8
WS	4.7	4.7	4.5	4.2	4.6	4.8	3.4	3.5
LR	1.4	1.2	1.1	1.3	1.6	1.7	1.2	1.2

### Feature importance

**Fig. 6:**
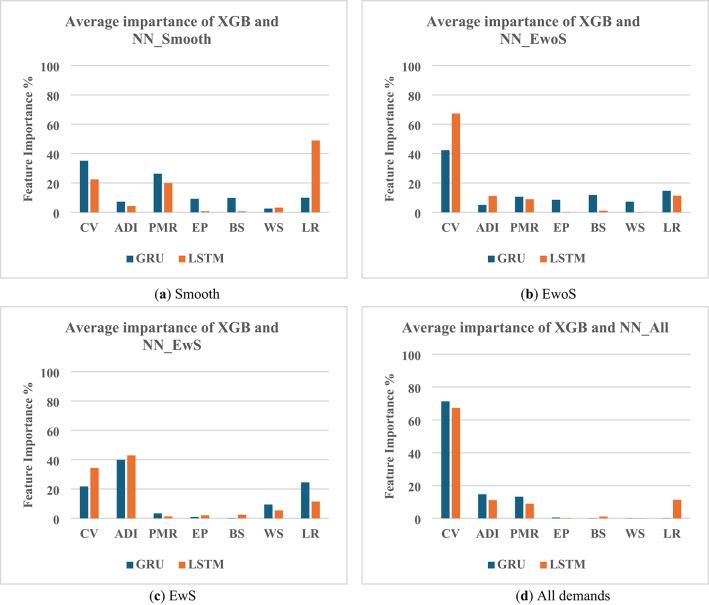
Average feature importance for different features depending on XGB and NN analyses for GRU and LSTM models for different demand situations (**a**) Smooth, (**b**) Erratic without Spikes, (**c**) Erratic with Spikes, and (**d**) All demand (DL hyperparameters show higher importance to the values of WMAPE% in feature importance than in linear correlations).

Since GRU and LSTM are deep learning models, non-linear relationships are also explored using XGBoost (XGB) and Neural Networks (NN) to assess feature importance. The results in Fig. 6 show the average importance calculated from both methods.

The analysis revealed that GRU hyperparameters exert a stronger non-linear influence on WMAPE% than initially indicated by linear methods, particularly in the context of erratic demand patterns. In smoother datasets, Batch Size (9.8%), Epochs (9.2%), and Learning Rate (9.9%) demonstrated moderate importance. However, as demand variability increased, the Learning Rate became significantly more influential, rising to 14.6% in the EwoS dataset and 24.5% in the EwS dataset. Similarly, the importance of Window Size grew from 2.5% in smooth datasets to 9.4% in EwS. When analyzing the combined dataset, the influence of data characteristics became more prominent, thereby diminishing the relative impact of individual hyperparameters. On the other hand, generally, the Epochs and Batch Size hyperparameters of LSTM show lower impacts compared to GRU. Slight importance appears to the value of the Window Size only in the case of EwS (~ 5.4%). As for the Learning Rate, its importance is higher in case of low DCI, Smooth demand (~ 49%), while its importance is almost constant (~ 11.5%) in other demand cases.

## Discussion

### Correlation analysis

From the above results, it can be seen that the overall complexity of demand has a direct linear correlation with the multi-period forecasting error. This is clear in cases where the sporadic spikes are not severe (smooth and EwoS cases), since learning from relatively complex, fluctuating demands is getting more challenging as the complexity increases. In addition, the results suggest that DL hyperparameters do not exhibit strong linear correlations with WMAPE%, indicating the likelihood of non-linear relationships. Furthermore, while ADI and PMR contribute to CV, ADI may have a more direct effect on WMAPE% in datasets characterized by spikes since these sporadic spikes could be significantly high non-zero demands at periods of promotions, for instance, or sudden zero demands in some periods. This points at the need to study the data properties collaboratively due to their interdependences, see Figs. 4 and 5, and Table 8, and separately for the importance of some properties in special demand data (spiky demands). The non-linear feature importance analysis shows higher correlations between forecast errors and the DL hyperparameters. This might be due to the varying ranges of the values of various hyperparameters and the necessity to adapt the combination of the hyperparameters to the data properties for better learning and higher accuracy.

### General analysis of the effects of different hyperparameters

While ML and DL methods can capture complex patterns, their performance heavily depends on proper hyperparameter tuning. This subsection analyzes both successful forecasts and failure cases, where the GRU and LSTM models predicted zero demand across all periods of the forecasting horizon, highlighted in Table 9. These failures reflect inadequate hyperparameter configurations and are excluded from the above main analysis but discussed separately, here, to guide practical avoidance. Since the forecasts of all periods are equal to zero, the resulting cumulative absolute error measured in WMAPE% has the value of 100% in all cases. As a brief discussion of these cases, some specific hyperparameter values show risks of higher failure probability. For instance, high Learning Rates of (0.1 and 0.05), Epochs of (80), small Batch Sizes (5), and small Window Size (10) are the most frequent hyperparameter values in most failure cases. These factors exhibit synergistic effects: the combination of high LR, small BS, and limited WS creates a multiplicative failure risk where noisy gradients, aggressive updates, and incomplete patterns compound to prevent the GRU and LSTM models from learning meaningful representations. The comparison between failure counts and percentages in GRU and LSTM methods suggests the following points. First, in contrast to the results in Fig. 3, which showed that the GRU results in lower WMAPE% values on average, the LSTM shows lower failure probability in all cases. Second, the failure probability increases with both methods as the demand complexity increases.

**Table 9:** Numbers and percents of successful and failed forecasting runs, for GRU and LSTM models, with all demand cases.

Metric	Smooth				EwoS				EwS				All			
	GRU		LSTM		GRU		LSTM		GRU		LSTM		GRU		LSTM	
	#	%	#	%	#	%	#	%	#	%	#	%	#	%	#	%
Total Rows	285	%	285	%	285	%	285	%	285	%	285	%	855	%	855	%
Failed Forecasts (WMAPE%=100%)	73	25.6	37	13	75	26.3	61	21.4	112	39.3	95	33.3	260	30.4	193	22.6
Successful Forecasts	212	74.4	248	87	210	73.7	224	78.6	173	60.7	190	66.7	595	69.6	662	77.4

The EwS scenario had the highest number of failed forecasts, highlighting the challenge of predicting spiky demand, even with DL models. Since each dataset’s properties remain constant, forecast success or failure is solely influenced by the hyperparameter combinations, which is the focus of the next subsection.

#### Hyperparameter comparisons of failed and successful cases for all data gathered

*GRU*.

**Fig. 7:**
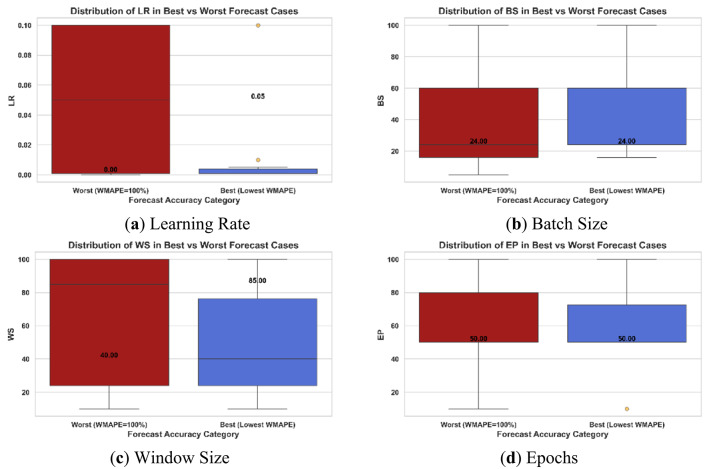
Box plots of hyperparameter values of (**a**) Learning Rate, (**b**) Batch Size, (**c**) Window Size, and (**d**) Epochs for the best and worst GRU forecasting cases of the All-Demands scenario, only as an example.

To guide practitioners in selecting effective hyperparameter values, this subsection compares the top and bottom 10% of WMAPE% results. This can help reduce reliance on exhaustive tuning methods like GridSearchCV or random search. Figure 7 illustrates the comparison for the combined dataset.

- Learning Rate: Crucial across all scenarios. Best cases consistently use low values (80% at LR = 0.001), while worst cases span a wide range. Slower learning appears to improve multi-period forecasting. The learning rate is the most crucial hyperparameter because it directly affects the stability of the recurrent network’s training. Very low values trap the model in weak initializations, leading to a limited range of stability, while high values can lead to gradient explosion during backpropagation with time, leading to divergence. Further, erratic demands with high complexity may intensify the sensitivity of the learning rate by introducing high gradient noise. Though Adam optimizer’s adaptive moments help lessen these effects, they cannot fully balance when the base learning rate is inadequately selected.
- Batch Size: A value equal to the forecasting horizon (24) appears in 50%–70% of best cases across all scenarios, suggesting it helps capture repeating patterns and align input-output timeframes.
- Window Size: Less impactful than LR, but trends vary. Low to moderate WS values perform better in EwoS and All scenarios; higher WS values are more effective in EwS. Effective WS range is 1–3 times the forecast horizon; larger windows may dilute short-term signals.
- Epochs: No strong effect observed, but very low values tend to reduce accuracy, likely due to underfitting.

After analyzing the failure VS best cases for each of the tackled demand types, Table 10 summarizes the recommended hyperparameter values in each case as taken from the best (lowest WMAPE%) 10% of the cases. First, Learning Rate (LR) is consistently low across all best cases, which is a strong indicator of stability. Batch Size (BS) and Window Size (WS) vary by scenario, suggesting scenario-specific tuning; according to the demand type, there might be a recommended range of their sizes. Finally, Epochs (EP) are generally moderate, avoiding both very low and very high values.

**Table 10:** Insights into the selection of GRU hyperparameters for different demand classes, drawn from minimum achieved WMAPE% cases.

Demand type	Epochs	Learning Rate	Batch size		Window Size
Smooth	A moderate value is better in all demand cases (50 to 80)	Relatively low values are better in all cases, specifically (0.001 and 0.005)	Not less than the forecasting horizon in all cases	1 to 2 times the forecasting horizon, 1 is more frequent	1 to 3 times the FH
EwoS				1 to 2 times the forecasting horizon	1 to 3 times the FH
EwS				BS = FH is the best value for better accuracy	2 to 4 times the FH (longer WS are more recommended)
All				1 to 2 times the forecasting horizon, 1 is more frequent	1 to 3 times the FH

*LSTM*

**Fig. 8:**
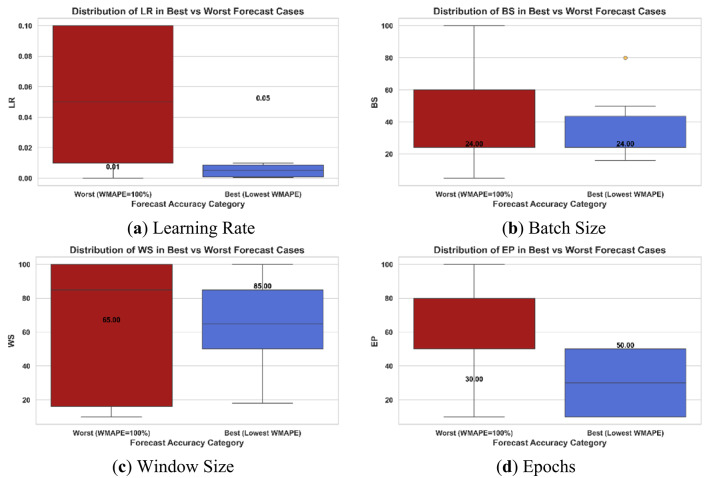
Box plots of hyperparameter values of (**a**) Learning Rate, (**b**) Batch Size, (**c**) Window Size, and (**d**) Epochs for the best and worst LSTM forecasting cases of the All-Demands scenario, only as an example.

As for LSTM, a smaller Learning rate value appears (0.0005) in the best cases, a lower number of epochs is recommended, and longer window sizes are recommended in the cases of erratic demands compared to GRU, as shown in Fig. 8.

Table 11 displays the recommended hyperparameter values that might increase the probability of lowering the WMAPE% values. It can be seen that lower epochs are generally recommended in smooth and EwoS demands; however, larger epochs are recommended in cases of spiky demands for better accuracy. As GRU, lower Learning Rates are better, and batch sizes are better to be equal to the length of the forecasting horizon or multiples of it to adapt the data-dependent relationship to the forecasting horizon’s length during learning.

**Table 11:** Insights into the selection of LSTM hyperparameters for different demand classes, drawn from the minimum achieved WMAPE% cases.

Demand type	Epochs	Learning Rate	Batch size		Window Size
Smooth	10 to 50	Relatively low values are better in all cases, specifically (0.0005, 0.001 and 0.005)	Not less than the forecasting horizon in all cases	1 to 2 times the forecasting horizon, 1 is more frequent	1 to 3 times the FH
EwoS	10 to 50			BS = FH is best value for better accuracy	1 to 4 times the FH
EwS	50 to 80			BS = FH is best value for better accuracy	4 times the FH (longer WS are more recommended)
All	10 to 50			1 to 2 times the forecasting horizon, 1 is more frequent	1 to 3 times the FH

### An integrated framework to recommend the hyperparameter values suitable for the data properties

To summarize the insights of this research for practical applications, an integrated Multi-output Random Forest framework is developed to enhance and accelerate the process of selecting hyperparameters for the DL model, based on the properties of the new dataset. As might be seen in the previous section, there are general guidelines to the selection of hyperparameters of DL methods; besides, there are hyperparameter recommendations that are specific to the method itself and its interactions with various demand complexity. This framework is a data-driven approach that relies on its decisions about hyperparameter selection for any DL method on data resulting from runs conducted only by the same method. In this section, the framework depends on the GRU method and GRU experimentation data only, as an example.

**Fig. 9:**
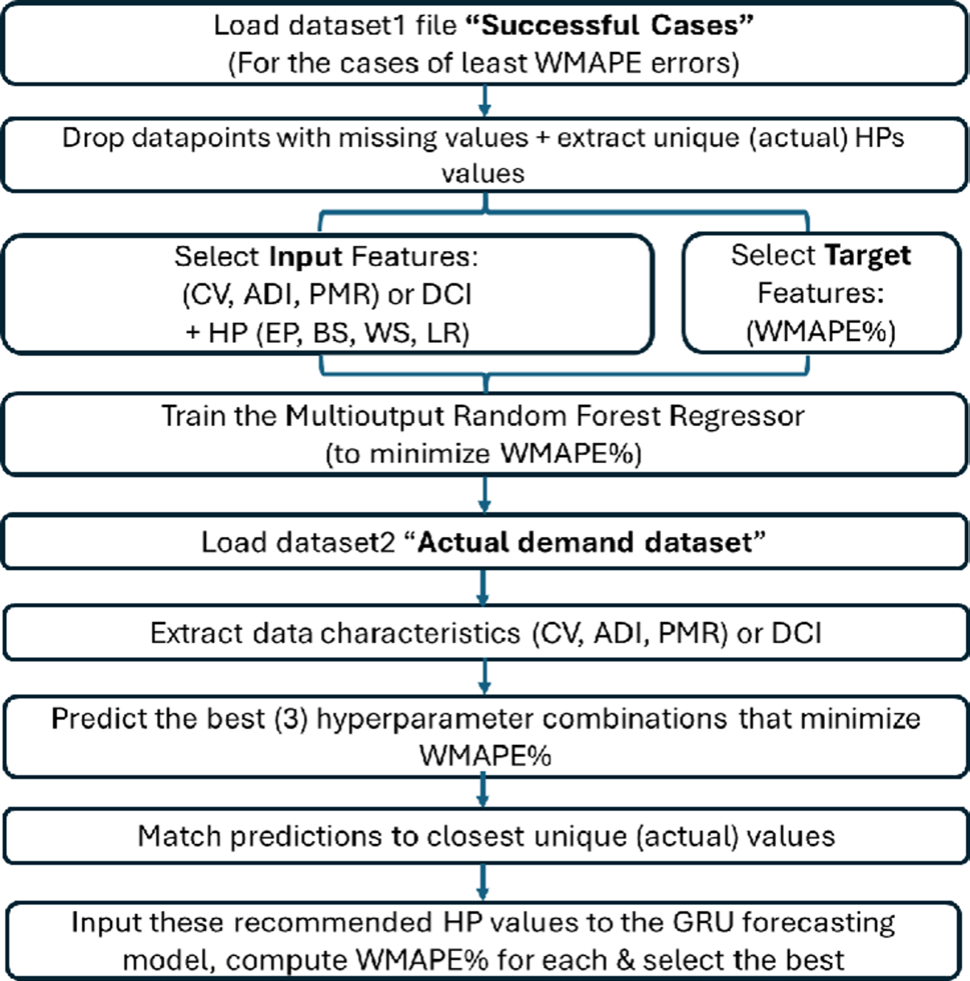
Integrated framework to select the hyperparameters’ values according to data characteristics.

The integrated framework employs a data-driven two-step forecasting approach. First, a Random Forest (RF) model predicts GRU hyperparameters, which are then used in the second step for forecasting. The RF training dataset is generated by running GRU on multiple time series with varying demand characteristics ((CV, ADI, PMR), or DCI) and hyperparameter combinations (EP, BS, WS, LR), recording WMAPE% for each run. After cleaning missing values, features are categorized into inputs (data characteristics and hyperparameters) and target features (WMAPE%). The RF model is trained to minimize WMAPE% using an 80/20 split, random seed (42), and 100 estimators. For new data, its characteristics are extracted, and RF predicts the best three hyperparameter combinations that might produce the three minimum WMAPE% values. The values of the hyperparameters of the predicted combinations are set equal to the closest actual unique values to avoid unrealistic scaling (e.g., EP = 24.73). These adjusted hyperparameter combinations are then applied in the GRU forecasting step, and the forecast resulting in the minimum WMAPE% value is selected. Figure 9 illustrates the framework.

As a comparison between the present framework and grid/random Search and Bayesian optimization, this framework has the following advantages:

1. Faster, since it predicts the suitable hyperparameters in seconds compared to larger times with other methods.

2. It learns from multiple forecasting experiments rather than independently optimizing each dataset, which leverages cross-dataset knowledge.

3. Provides immediate hyperparameter recommendations for totally new datasets.

4. Relies upon interpretable data characteristics ((CV, ADI, PMR), or DCI), which are meaningful in the demand forecasting context.

To test the applicability of the integrated framework, six generated datasets were used, each two of which represent one of the considered demand cases: smooth (test1 and test2), EwoS (test3 and test4), and EwS (test5 and test6). The characteristics of the test datasets is shown below in Table 12.

**Table 12:** Statistics of the test sets used to validate the integrated framework.

Dataset	Count	Min	Max	Mean	Std Dev	CV	ADI	PMR	DCI
Test1	1500	0	80	33.67	15.97	0.47	1.029	2.38	0.096
Test2	1650	0	78	24.60	16.74	0.68	1.163	3.17	0.241
Test3	1500	0	200	47.14	35.34	0.75	1.198	4.24	0.289
Test4	1800	0	47	6.00	5.24	0.87	1.076	7.83	0.283
Test5	1800	0	73	5.83	5.16	0.88	1.068	12.52	0.314
Test6	1800	0	69	3.99	4.13	1.04	1.124	17.30	0.425

**Fig. 10:**
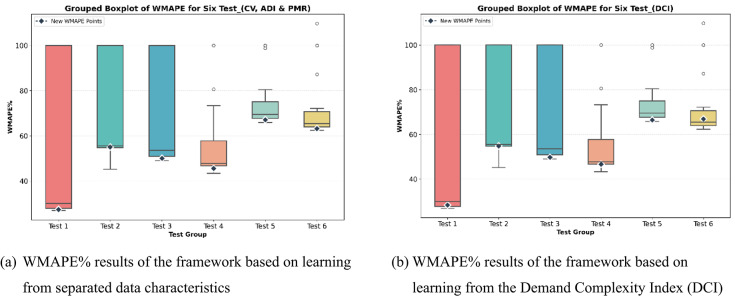
Positioning of the WMAPE% values resulting from the integrated framework in the DOE solution spaces of the Test datasets, for two framework configurations (**a**) learning from separated demand characteristics (CV, ADI, and PMR), and (**b**) learning directly from the DCI.

To address the results of applying the integrated framework on the test sets, the DOE configurations of the hyperparameters are applied to them, and the resulting WMAPE% is recorded, just to position the integrated framework’s resulting WMAPE%. It is to be noted that this DOE data of the test set is unseen by the integrated framework. In addition, to validate the usage of the DCI to express the complexity of demands, the learning dataset fed to the RF step of the framework was based on separated data characteristics metrics (CV, ADI, and PMR), and another time based on DCI to compare results. The box-plots of the WMAPE% values of the test data sets and the WMAPE% resulting from the integrated framework are all shown in Fig. 10. The figures show that in both cases, separated characteristics and DCI, the framework resulted in reliable forecasting accuracies.

The detailed WMAPE% values of all cases are shown in Table 13. It is seen that both cases result in WMAPE% values below the median WMAPE% in all cases, except for the case of Test6, which might be due to its high PMR value.

**Table 13:** Resulting WMAPE% values, and their corresponding deciles in the full DOE experimentation results, after using the integrated framework, when learning from separated demand characteristics and the DCI.

	Min	Max	Mean	Median	Separated (CV + ADI + PMR)		DCI	
					WMAPE %	Decile	WMAPE %	Decile
Test1	26.91	100	51.82	30.04	27.35	0–1	28.36	3–4
Test2	45.18	100	69.39	55.57	54.97	3–4	54.82	2–3
Test3	49.01	100	67.97	53.57	50	0–1	49.77	0–1
Test4	43.33	100	58.62	47.78	45.46	1–2	46.67	2–3
Test5	65.85	100	75.51	69.51	67.07	0–1	66.46	0–1
Test6	62.41	109.77	72.95	65.41	63.16	0–1	66.92	6–7

To illustrate the computational efficiency of the proposed framework, we compared the time required to run the GRU model for all hyperparameter combinations in the DOE with the time taken by the integrated framework using a sample dataset (Test5). The average runtime for a single GRU execution was 5 min and 5 s. Running all (57) DOE combinations required 4 h, 49 min, and 44 s, whereas the integrated framework completed the task in just 16 min and 40 s—including three GRU runs for the selected combinations—while achieving a WMAPE% within the first decile. This represents only 5.8% of the time needed for the DOE runs, which themselves cover just a subset of the hyperparameter space that GridSearchCV, for instance, would explore for the same parameter values.

The results obtained in the present work are subject to the following limitations. The analysis focuses on three demand types—smooth, erratic without spikes, and erratic with spikes. All results are obtained for a fixed forecasting horizon of 24 periods. The results obtained are due to the use of two DL methods, namely, GRU and LSTM, only with their adopted architectures.

## Conclusion

This study explored the impact of GRU and LSTM hyperparameters and demand data characteristics on the accuracy of multi-period demand forecasts using deep learning. By analyzing three types of demand datasets, smooth, erratic without spikes, and erratic with spikes, the research provided insights into how data variability and model configuration influence forecasting performance. For instance, demand complexity significantly influences forecasting accuracy, with WMAPE increasing by up to 14.6% per unit rise in DCI for GRU and 11.3% for LSTM, highlighting the need for complexity-driven model optimization.

The results showed that data properties, particularly Coefficient of Variation (CV) and Peak-to-Mean Ratio (PMR), are the most influential factors affecting forecast accuracy. Among the GRU and LSTM hyperparameters, Learning Rate emerged as the most critical, with low values consistently associated with better performance. Other hyperparameters, such as Batch Size and Window Size, also showed notable effects, especially when aligned with the forecasting horizon.

Failure cases, especially in spiky demand scenarios, highlighted the sensitivity of DL (GRU and LSTM) models to hyperparameter configurations. Comparing the best and worst performing cases revealed practical guidelines for selecting initial hyperparameter values, potentially reducing the need for exhaustive tuning. According to the experimental.

The use of WMAPE% as the accuracy metric proved effective in handling zero-demand periods and normalizing results across datasets. The combination of linear correlation and feature importance analyses, including XGBoost and Neural Network to capture non-linear relationships, provided a comprehensive understanding of the relationships between features and forecast accuracy.

This study makes three key contributions. First, it provides a targeted analysis of how data characteristics interact with deep learning hyperparameters, offering deeper insights into the forecasting challenge. Second, we introduce the Demand Complexity Index (DCI), a normalized metric that consolidates demand variability (CV), zero-demand frequency (ADI), and peak severity (PMR). The DCI demonstrates strong correlations with WMAPE% across all demand types, with correlation strength increasing as DCI decreases. Finally, we propose a data-driven, multi-output forecasting framework capable of predicting hyperparameter configurations that yield high accuracy based on demand characteristics, while significantly reducing computation time and cost.

## Future work

While this study focused on GRU and LSTM models, the analytical framework can be extended to other DL architectures and forecasting domains. Future research may explore automated hyperparameter optimization techniques and the integration of external factors (e.g., promotions, seasonality) to further enhance forecast accuracy. Besides, discussing the suitability of the planning horizon’s length for the forecasting problem might be an interesting topic.

## Data Availability

The resulting experimental data is published on (Zenodo) repository, under the Creative Commons Attribution 4.0 International license (CC-BY-4.0), with the following persistent DOI: [https://doi.org/10.5281/zenodo.17574846]
